# Optimization of OPEFB lignocellulose transformation process through ionic liquid [TEA][HSO_4_] based pretreatment

**DOI:** 10.1038/s41598-021-90891-3

**Published:** 2021-05-31

**Authors:** Muhammad Nurdin, Haznan Abimanyu, Hadijah Putriani, L. O. M. Idal Setiawan, Maulidiyah Maulidiyah, Dwiprayogo Wibowo, Ansharullah Ansharullah, Muh. Natsir, La Ode Agus Salim, Zul Arham, Faizal Mustapa

**Affiliations:** 1 Department of Chemistry, Faculty of Mathematics and Natural Sciences, Universitas Halu Oleo, Kendari, 93231 Southeast Sulawesi Indonesia; 2 Research Center for Chemistry, Indonesian Institute of Sciences (LIPI), Kawasan PUSPIPTEK, Serpong, 15314 Tangerang Selatan Indonesia; 3 Department of Environmental Engineering, Faculty Engineering, Universitas Muhammadiyah Kendari, Kendari, 93117 Southeast Sulawesi Indonesia; 4 Department of Food Science & Technology, Faculty of Agriculture, Universitas Halu Oleo, Kendari, 93231 Southeast Sulawesi Indonesia; 5 Department of Mathematics and Natural Science, Institut Agama Islam Negeri Kendari, Kendari, 93116 Southeast Sulawesi Indonesia; 6 Department of Aquaqulture, Faculty of Sciences and Technology, Institut Teknologi dan Kesehatan Avicenna, Kendari, 93116 Southeast Sulawesi Indonesia

**Keywords:** Analytical chemistry, Ionic liquids, Energy

## Abstract

Research on the transformation of Oil Palm Empty Fruit Bunches (OPEFB) through pretreatment process using ionic liquid triethylammonium hydrogen sulphate (IL [TEA][HSO_4_]) was completed. The stages of the transformation process carried out were the synthesis of IL with the one-spot method, optimization of IL composition and pretreatment temperature, and IL recovery. The success of the IL synthesis stage was analyzed by FTIR, H-NMR and TGA. Based on the results obtained, it showed that IL [TEA][HSO_4_] was successfully synthesized. This was indicated by the presence of IR absorption at 1/λ = 2814.97 cm^−1^, 1401.07 cm^−1^, 1233.30 cm^−1^ and 847.92 cm^−1^ which were functional groups for NH, CH_3_, CN and SO_2_, respectively. These results were supported by H-NMR data at δ (ppm) = 1.217–1.236 (N–CH_2_–CH_3_), 3.005–3.023 (–H), 3.427–3.445 (N–H^+^) and 3.867 (N^+^H_3_). The TGA results showed that the melting point and decomposition temperature of the IL were 49 °C and 274.3 °C, respectively. Based on pretreatment optimization, it showed that the best IL composition for cellulose production was 85 wt%. Meanwhile, temperature optimization showed that the best temperature was 120 °C. In these two optimum conditions, the cellulose content was obtained at 45.84 wt%. Testing of IL [TEA][HSO_4_] recovery performance for reuse has shown promising results. During the pretreatment process, IL [TEA][HSO_4_] recovery effectively increased the cellulose content of OPEFB to 29.13 wt% and decreased the lignin content to 32.57%. The success of the recovery process is indicated by the increasing density properties of IL [TEA][HSO_4_]. This increase occurs when using a temperature of 80–100 °C. The overall conditions obtained from this work suggest that IL [TEA][HSO_4_] was effective during the transformation process of OPEFB into cellulose. This shows the potential of IL [TEA][HSO_4_] in the future in the renewable energy sector.

## Introduction

**Figure 1:**
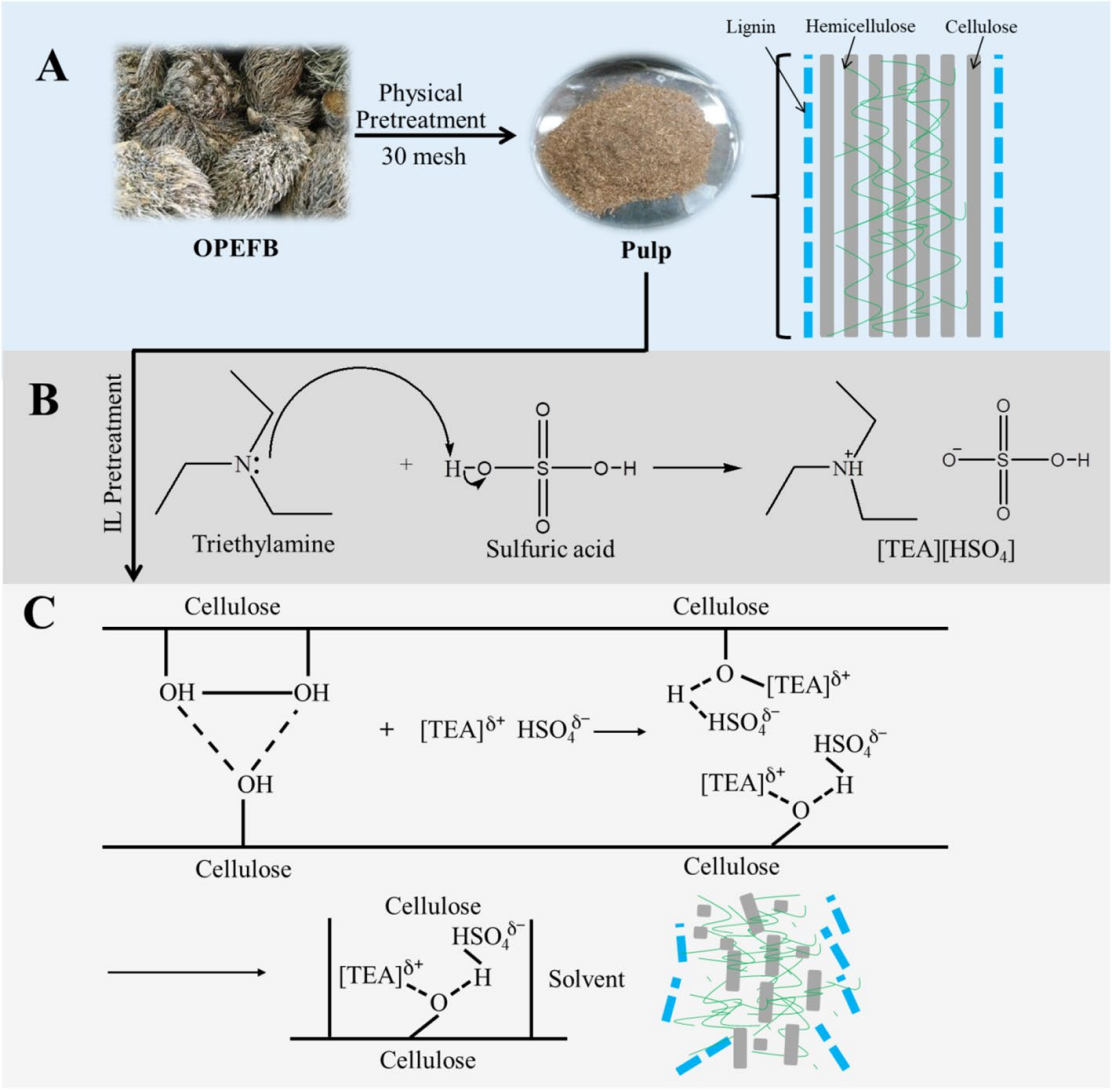
(**A**) Protection of cellulose by lignin and hemicellulose, (**B**) mechanism of IL [TEA][HSO_4_] synthesis reaction, and (**C**) mechanism of the IL during pretreatment of OPEFB.

The transformation of OPEFB waste as a raw material for renewable energy sources, especially in bioethanol production, has still been the concern of many researchers. In general, the biomass transformation process consists of several important stages such as pretreatment and fermentation^1–3^. Although the cellulose content in OPEFB was reported to be high, ranging from ± 27–50%, the presence of lignin and hemicellulose was a problem in the transformation process. This problem is the main focus of the pretreatment process^4–6^. The lignin and hemicellulose contents in OPEFB were reported to range from 18–35% and 24–35%, respectively. In addition to inhibiting the catalysts action, the content of lignin and hemicellulose also interferes with the activity of cellulose enzymes in converting glucose into bioethanol. Lignin and hemicellulose will protect cellulose through the mechanism of forming a dense surface structure, so that the catalyst and cellulose enzymes do not work optimally. An illustration that explains this situation can be seen in Fig. 1.

In overcoming the pretreatment problem of OPEFB, many pretreatment methods have reported their performance. Pretreatment is an important step in opening or stretching the lignocellulose structure. Pretreatment can be done either physically, chemically or a combination of the both. Physical methods such as uncatalyzed steam-explosion^7,8^, liquid hot water (LHW)^9,10^, mechanical communication^11^, and high energy radiation^12^. Meanwhile, Chemical methods such as catalyzed steam-explosion^13^, acid pretreatment^14,15^, alkaline pretreatment^16,17^, ammonia fiber/freeze explosion (AFEX)^18,19^, organosolv^20,21^, and pH-controlled liquid hot water^10^. To improve pretreatment results, a combination of these two methods is often carried out such as the combination of the hydrothermal method with sulfuric acid. Although this method can improve the purity of cellulose and enzyme performance during the bioethanol production process, however these methods have also an impact on the high use of hazardous chemical solvents during the pretreatment process which can cause new environmental problems^22^. Another impact that has also been reported is how pretreatment such as physical pretreatment can affect the properties of cellulose^23^. Based on these problems, OPEFB pretreatment was mostly directed at the Ionic Liquid (IL) based pretreatment system. This system is reported to be an environmentally friendly biomass pretreatment system with a low amount of solvent.

IL pretreatment is a new system for the development of the OPEFB pretreatment method. This method is more environmentally friendly, effectively destroys lignin and hemicellulose bonds without damaging the glucose structure, effectively reduces cellulose crystallinity, low volatility and vapor pressure, and can be recycled^24,25^. In addition, IL has special properties such as wider fluid temperatures, high thermal stability, and negligible vapor pressure^26^. Certainly, these essential properties are needed in the lignocellulose biomass transformation. IL has been reported for biomass pretreatment among others 1-buthyl-3-methylpyridinium chloride, [Bmpy][Cl]^27^; 1-ethyl-3-methylimidazolium acetate, [Emim][OAc]^28^; 1-ethyl-3-methylimidazolium diethyl phosphate, [Emim][DEPO_4_]^29^; 1,3-dimethylimidazolium methyl sulfate, [Dmim][MeSO_4_]^29^; 1-N-ethylimidazolium chloride, [C_2_mim][Cl]; 1-N-buthylimidazolium chloride, [C4mim][Cl]; 1-butyl-3-methylimidazolium chloride, [BMIM][Cl]^30,31^, 1-allyl-3-methylimidazolium chloride, [Amim][Cl]; N-methyl-N-methylimidazolium dimethyl phosphate, [Mmim][DMP], 1-butyl-3-methylimidazolium bis (trifluoromethylsulfony), [BMIM][NTf_2_]^32–34^ and 1-butyl-3-methylimidazolium acetate, [BMIM][OAc]^31^. However, some ILs show shortcomings for biomass pretreatment applications. For example, [Emim][OAc] application requires high cost, low thermal stability, low humidity tolerance, and in dissolving cellulose requires a low moisture content, this is in contrast to the high moisture content of the biomass.

As an alternative to the deficiency of IL, it is important to choose the type of anion. In this regard, the synthesis and application of triethylammonium hydrogen sulphate [TEA][HSO_4_] as IL for the pretreatment process resulted in lower production costs^30^. In addition, the length of the alkyl chain in ammonium plays an important role in the effectiveness of biomass pretreatment. Besides price, another important consideration of the broad application of IL [TEA][HSO_4_] to pretreatment processes is recovery and recycling^24^. Based on this, we specifically reported IL [TEA][HSO_4_] activity for the transformation of OPEFB. The IL synthesis stage was carried out using the one-pot method by combining triethylammonium and sulfuric acid. In the pretreatment stage, we optimized the performance of IL [TEA][HSO_4_] based on concentration and temperature. The recycling process is carried out in several stages, including filtering, mixing solvents, washing and separating. The purpose of our recycling process is to optimize the use of IL [TEA][HSO_4_].

Although our concern in this work is the transformation of OPEFB for bioethanol production, however, in the development of other renewable technologies, the pretreatment results obtained can be expanded in application such as for the manufacture of activated carbon and carbon nanotubes. Two areas of study have been reported by^35^. In addition, the results of OPEFB pretreatment can be used as ingredients to decompose food waste previously reported by^36^.

In general, the use of various types of IL in the bioethanol production stage shows good performance. The use of IL is effective in increasing the enzymatic hydrolysis of cellulose to glucose. Imidazolium-based IL is reported to increase porosity and specific surface area accessible to enzymes during hydrolysis and fermentation. The use of this type of IL resulted in a glucose content of 97.7%^37^. Another IL type that has been reported is pyrrolidonium based IL. Its use causes the enzymatic hydrolysis process to effectively produce a glucose content of 91.81%^38^. The application of amino acid-based IL was also reported to be effective in producing a glucose content of 87.7%^39^. Based on these results it is known that the application of IL in bioethanol production is influenced by the use of the IL composition and temperature during the pretreatment process.

## Experimental methods

### Apparatus and materials

Starting materials for synthesizing IL [TEA][HSO_4_] are H_2_SO_4_ 97%, ethanol 97%, triethylamine and potassium carbonate were purchased from Sigma Aldrich and the apparatus used from Iwaki Pyrex. The characterization was measured by Dr. Haznan Abimanyu in Research Center for Chemistry, Indonesian Institute of Sciences by using FTIR (Nicolet FT-IR iS10, USA). The ^1^H-NMR was recorded on a Bruker 500 MHz spectrometer. Chemical shifts (δ) are reported in ppm with the D_2_O peak at 8.0 ppm and TGA (SDT-2960, USA) was analyzed to observe temperature rate towards % weight loss of IL [TEA][HSO_4_]. Other apparatus are vacuum oven (Manufacturers GT-BM12), magnetic stirrer (DLAB Classic MS-H-S), rotary evaporator (DLAB Led RE100-S).

### Synthesis of IL [TEA][HSO_4_]

Synthesis of IL [TEA][HSO_4_] has been conducted by referring to the method reported by^40^. In summary, 75.9 g (750 mmol) of triethylamine was inserted into a three-neck flask and stirred by using a magnetic stirrer under cold conditions. Under stirring, it slowly added 5 M H_2_SO_4_ 97% and ethanol solution (1:5 w/w) for 24 h. After that, the ethanol solution was removed using a vacuum evaporator for 2 h and dried in a vacuum oven at 40 °C for 8 h. Finally, the IL [TEA][HSO_4_] was analyzed using FTIR, ^1^H-NMR and TGA.

### Preparation of OPEFB pulp

The pretreatment process in this work begins with the preparation of pulp from the OPEFB. OPEFB raw materials obtained from PTPN-VII (Lampung-Indonesia) were milled and separated using a 30 mesh sieve. The separated pulp was dried in the oven for 24 h until the moisture content was below 10 wt%. Furthermore, the lignocellulose content, moisture and ash content of the pulp were analyzed using standard procedures from the National Renewable Energy Laboratory (NREL).

### OPEFB biomass pretreatment

The IL [TEA][HSO_4_] based OPEFB pretreatment process was carried out by studying two variations, including the IL composition and temperature. The IL compositions (wt%) studied were 80%, 85% and 91%. The determination of this composition refers to Eq. (1), where this composition is the ratio between IL [TEA][HSO_4_] and deionized water, HACH 272-56 (IL [TEA][HSO_4_] : H_2_O). Meanwhile, the temperature variations (^o^C) studied were 30, 50, 80, 100, 120 and 150. In summary, IL [TEA][HSO_4_] and H_2_O were weighed as much as 8.0 g and 2.0 g, respectively. Both are put into the Schott bottle slowly and then homogenized. After that, the OPEFB pulp was weighed as much as 2.0 g and put into the Schott bottle. This composition was homogenized with a magnetic stirrer for 17 h at 80 °C using hot medium silicone oil. Furthermore, the filtrate is separated using a vacuum pump with the addition of methanol, while the resulting residue is prepared for analysis of the amount of lignocellulose after transformation. The same treatment was also used for the variation in the composition of 85% and 91%. The fundamental difference of this composition is the mass of IL [TEA][HSO_4_] used. The masses of IL [TEA] [HSO_4_] used for the 85% and 91% compositions were 10.0 g and 20.0 g, respectively. Meanwhile, the H_2_O mass and OPEFB pulp were kept constant. Every treatment, both composition and temperature variations, we do in duplicate.

1 $$ \% IL \left[ {TEA} \right]\left[ {HSO4} \right] = \frac{{mass of IL \left[ {TEA} \right]\left[ {HSO4} \right], wt}}{{ total mass \left( {\left[ {TEA} \right]\left[ {HSO4} \right] + H2O} \right), wt}} \times 100\% $$

### IL [TEA][HSO_4_] recovery

The recovery process is carried out on IL [TEA][HSO_4_] waste originating from the OPEFB pretreatment process. In summary, the IL [TEA][HSO_4_] waste was filtered on a vacuum filter with the addition of a methanol solution. To obtain IL [TEA][HSO_4_], the solution mixture consisting of IL [TEA][HSO_4_]-methanol is separated again on a rotary evaporator with a heating temperature of < 80 °C, then followed by washing IL [TEA][HSO_4_] using aquadest and centrifugation for 15 min at 4 °C at 1000 rpm. This process aims to separate IL [TEA][HSO_4_] and dissolved lignin during the OPEFB pretreatment process. Washing IL [TEA][HSO_4_] was carried out 2–3 times using 40 mL aquadest. IL [TEA][HSO_4_] which was still mixed with water was separated using a rotary evaporator at 100 °C.

### Cellulose and hemicellulose analysis by HPLC

A total of 0.30 g of dry sample measuring < 1 mm was put into the test tube, 3 mL of 72% H_2_SO_4_ was added, then the first stage was hydrolyzed for 1 h using an incubator at 30 °C. While homogenized every 15 min using vortex. The hydrosylate was transferred to a Schott bottle containing 87 mL of DI water. Subsequently, the sample was hydrolyzed in a second stage for 1 h in a vacuum autoclave at a temperature of 121 °C. Cooled to room temperature, filtered with a bunchner filter and 0.45 μm filter paper. The filtrate is collected and neutralized with CaCO_3_ to a neutral pH, then filtered with a 0.2 μm pore size filter using a syringe, and stored in autosamplervial HPLC. Furthermore, the cellulose and hemicellulose contents were analyzed using HPLC with an aminex HPX 87H column (300 × 7.8 mm) at a temperature of 65 °C with a mobile phase of 5 mM H_2_SO_4_ and a flow rate of 0.6 mL min^−2^ and a refractory index detector (waters 2414 T: 40 °C). Cellulose content is calculated by the equation:

2 $$ C_{GS} = \frac{Hs}{{H_{std} }} \times C_{stdG} $$

3 $$ \% Glucose = \frac{{C_{GS} x 87 ml/100}}{{W_{s} }} \times 100\% $$

4 $$ \% Celullose = \% glucose \times 0.9 $$

Note:

C_GS_: [glucose].

H_S_: Sample peak height.

H_std_: Standart peak height.

C_stdG_: [glucose standart].

W_S_: Dry sample weight.

While hemicellulose levels are calculated by the equation:

5 $$ C_{XS} = \frac{Hs}{{H_{std} }} \times C_{stdX} $$

6 $$ \% Xylose = \frac{{C_{XS} \times 87 ml/100}}{{W_{s} }} \times 100\% $$

7 $$ \% Hemicellulose = \% xylose \times 0.88 $$

Note:

C_XS_: [xylose].

H_S_: Sample peak height.

H_std_: Standart peak height.

C_stdX_: [xylose standart].

W_S_: Dry sample weight.

### Lignin analysis by UV–Vis

0.45 μm filter paper is weighed, and used to filter the sample through a vacuum filter system. The filter paper and the residue stuck on it are dried and weighed. Then the residue is ignited at 575 °C for 3 h using a furnace, and followed by weighing the weight of the ash obtained. As for the filtered filtrate, 0.03 mL was taken and put into a test tube containing 2.70 mL 4% H_2_SO_4_. Furthermore, the absorption was measured using a UV–Vis spectrophotometer at a wavelength of 205 nm. The dissolved and insoluble lignin levels are calculated using the equation:

8 $$ {\text{\% AIL}} = \frac{{W_{KS} - W_{K} - A}}{{W_{S} }} \times 100\% $$

9 $$ {\text{\% ASL}} = \frac{{\left( {Abs \times df/110} \right) \times \left( {87/1000} \right) }}{{W_{S} }} \times 100\% $$

10 $$ {\text{Levels }}\;{\text{of}} \;{\text{lignin}}\; {\text{total}} = \% AIL + \% ASL $$

Note:

%AIL: Acid insoluble lignin.

%ASL: Acid soluble lignin.

W_KS_: Weight of filter paper and samples after drying.

W: Weight of filter paper.

A: Ash weight.

Abs: Absorbance.

df: The dilution factor.

Ws: Dry sample weight (without moisture content).

## Results and discussion

### FTIR, H-NMR and TGA analysis

**Figure 2:**
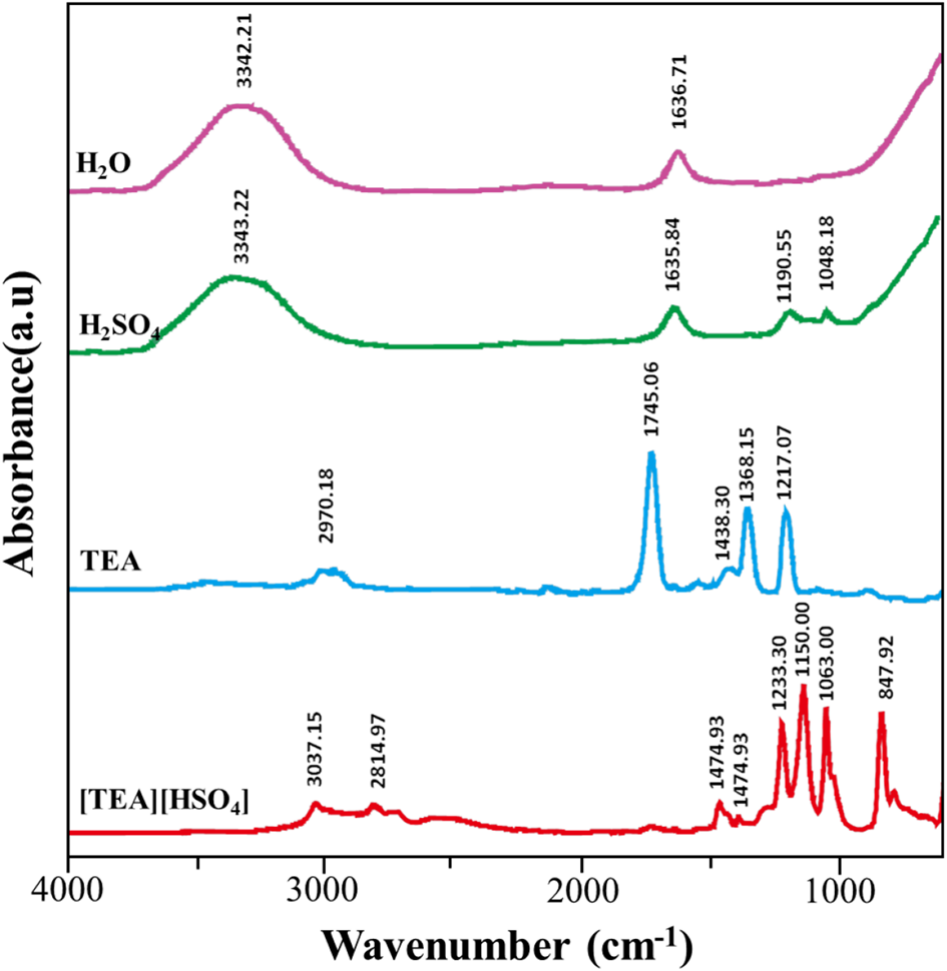
IR spectra of IL [TEA][HSO_4_] constituents.

The success in the synthesis process of IL [TEA][HSO_4_] was studied based on the results of FTIR, H-NMR and TGA analysis with literature comparisons. Figure 2 is the result of FTIR analysis of IL [TEA][HSO_4_] (red line) and its components include TEA (blue line), H_2_SO_4_ (green line) and H_2_O (purple line). Based on the IR spectra, we observed the appearance of new spectra at wave numbers of 2814.97 cm^−1^, 1401.07 cm^−1^, 1233.30 cm^−1^ and 847.92 cm^−1^. The results of the literature study show that the values of the wave numbers refer to the NH, CH_3_, CN and SO_2_ groups, respectively^41^. In addition, the success of the IL [TEA][HSO_4_] synthesis process was confirmed by the shift in the absorption peak for wave number 1438 cm^−1^ to 1474.93 cm^−1^ which characterized the CH_2_ functional group, as well as the shift in wave number 1048.18 cm^−1^ to 1063.00 cm^−1^ which characterizes the SO functional group.

**Figure 3:**
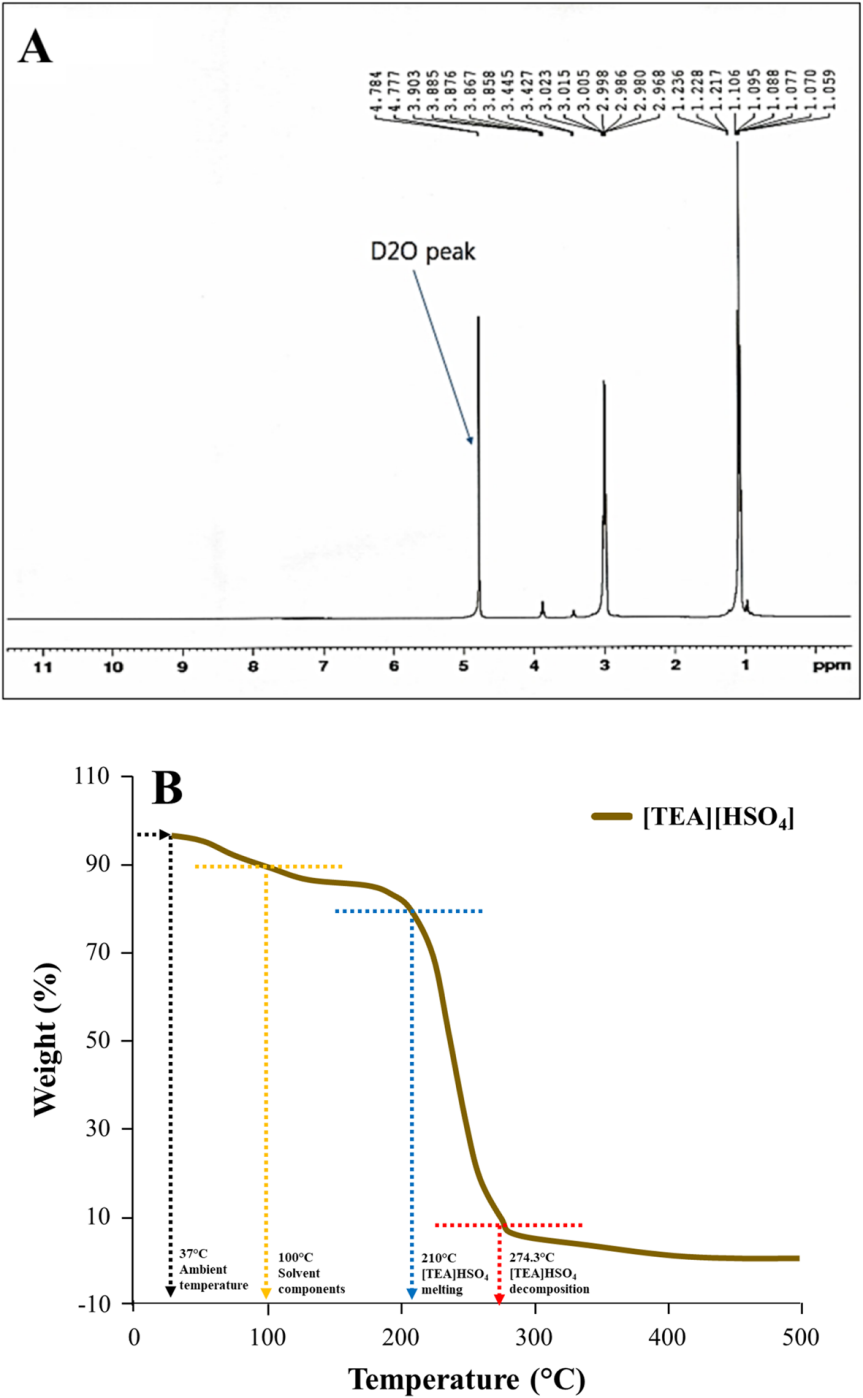
The H-NMR spectra (**A**) and thermal analysis (**B**) of IL [TEA][HSO_4_].

Figure 3A is an H-NMR spectra of IL [TEA][HSO4]. Based on the literature approach, there are similarities in the chemical shift values, δ (ppm) with those reported by^40,41^ at δ = 1.217–1.236 (N–CH_2_–CH_3_), 3.005–3.023 (–H), 3.427–3.445 (N–H^+^) and 3.867 (N^+^H_3_). The similarity in chemical shift values characterizes the success of the IL [TEA][HSO_4_] synthesis process based on the one-spot method. Another chemical shift data that confirms the success of the synthesis process occurs at δ = 1.106 (CH_2_–N). Figure 3B shows the results of the TGA analysis of IL [TEA][HSO_4_]. These results provide an overview of the physical properties of IL [TEA][HSO_4_] based on temperature. As a first step, we analyzed it under ambient temperature at 37 °C. Then there was a decrease in the percentage by weight from 37 to 100 °C which indicates that the solvent components have evaporated such as H_2_O and ethanol. From 100 to 210 °C illustrates the role of IL [TEA][HSO_4_] which is stable at high temperatures, this condition can be used as a variable in the pretreatment process. Another result we got was that IL [TEA][HSO_4_] was decomposed at a temperature of 274.3 °C to 500 °C.

### OPEFB pretreatment using IL [TEA][HSO_4_]

In the OPEFB pretreatment process using IL [TEA][HSO_4_], we optimized 2 important parameters, namely composition and temperature. Optimization of the composition is important because it is related to the amount of IL [TEA][HSO_4_] used and the increase in the amount of cellulose. In this work, the selection of the lowest percentage of IL [TEA][HSO_4_] composition of 80% was carried out with the consideration that the performance of IL [TEA][HSO_4_] will be affected by the number of water molecules. This effect is seen when the percentage of water molecules is smaller or greater than 20%, where the IL [TEA][HSO_4_] activity decreases^24^. So that every composition we propose always keeps the water percentage at 20%.

The mechanism of action of IL [TEA][HSO_4_] in OPEFB pretreatment can be seen in Fig. 1C. In the OPEFB pretreatment, IL [TEA][HSO_4_] will break down the hydrogen bonds of cellulose both intra and intermolecules, thereby facilitating the process of dissolving cellulose. As with other IL applications, cations and anions from IL [TEA][HSO_4_] play an important role in cellulose dissolution. Especially for the [HSO_4_]^−^ anion, this ion acts as a hydrogen bond acceptor for cellulose dissolution. When the OPEFB lignocellulose component was dissolved in IL [TEA][HSO_4_], the tissue inside the cell wall will be disturbed thereby reducing the recalcitrant nature of the OPEFB biomass. Cellulose which regenerates after pretreatment tends to be more amorphous in its macro structure making it easier for enzyme hydrolysis.

**Figure 4:**
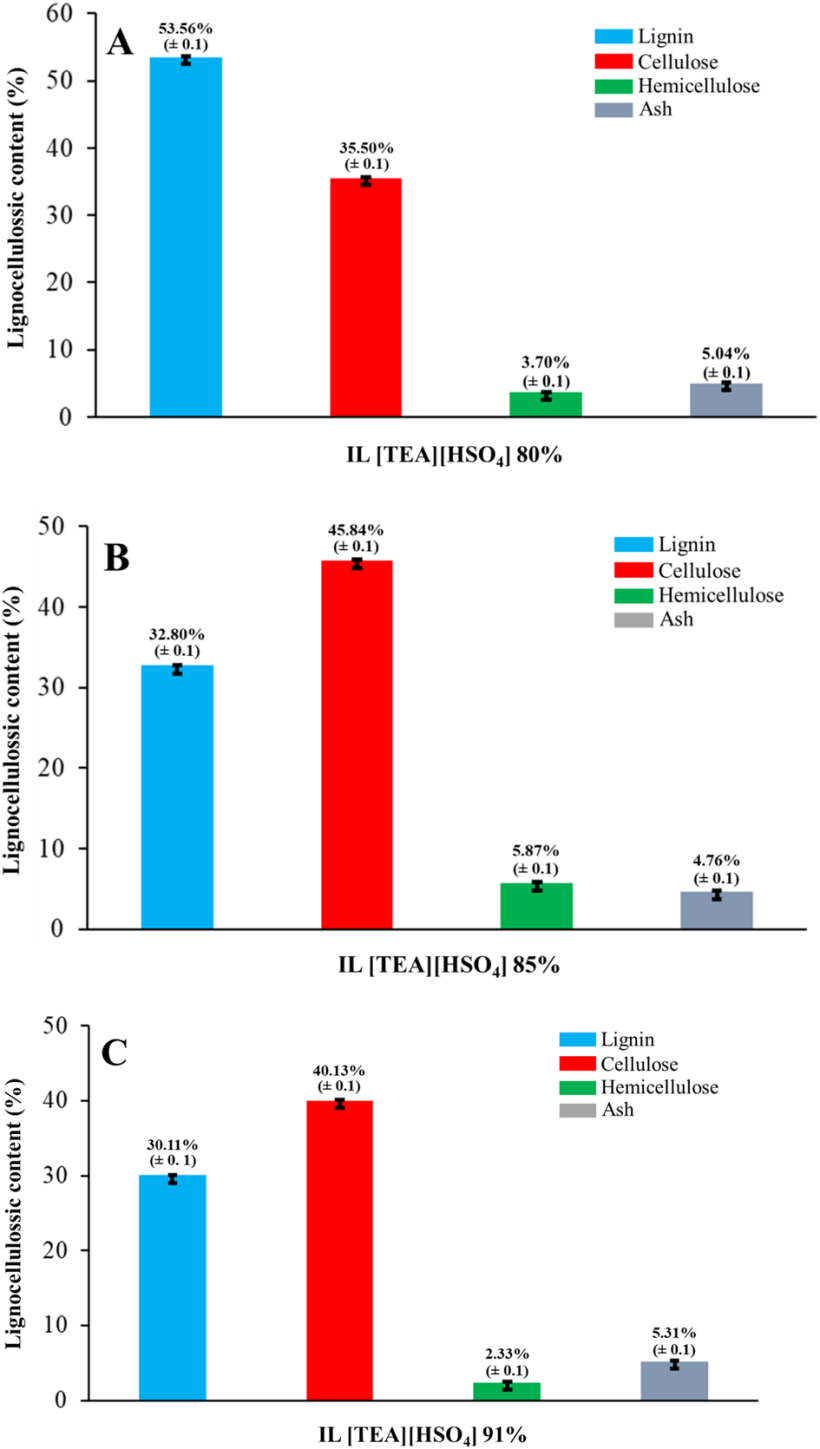
Optimization of the composition of IL [TEA][HSO_4_] for OPEFB pretreatment: (**A**) 80%, (**B**) 85% and (**C**) 91%.

Figure 4 shows the optimization results of the IL [TEA][HSO_4_] composition for OPEFB transformation. We have observed the success of the OPEFB transformation through an increase in cellulose content. When comparing the data on lignocellulose content before pretreatment (Table 1), we found that there was a change in the percentage content of lignocellulose in both cellulose, hemicellulose and lignin in each IL [TEA][HSO_4_] composition tested. This shows that there is a mass effect of IL [TEA][HSO_4_] on the pretreatment of OPEFB.

**Table 1:** Lignocellulose content of OPEFB before pretreatment.

OPEFB	Content (%)	
	Before pretreatment	After pretreatment
Cellulose	27.40 ± 0.01	45.84 ± 0.1
Hemicellulose	12.87 ± 0.01	5.87 ± 0.1
Lignin	34.61 ± 0.01	32.8 ± 0.1
Ash	3.02 ± 0.00	4.76 ± 0.1
Moisture content	2.80 ± 0.01	2.92 ± 0.1

The optimum pretreatment process occurred at the use of 85% composition (Fig. 4B), where in this composition there was an increase in cellulose content to 45.84% and a decrease in lignin and hemicellulose content to 32.80% and 5.87%, respectively. The same trend also occurred for the composition of 80% and 91%. However, the two compositions showed that the pretreatment rate of OPEFB to increase the cellulose content tended to be slower. At the 80% composition (Fig. 4A) the cellulose content only increased to 32.80%, while at 91% composition (Fig. 4C) the cellulose content only increased to 40.13%. The decrease in the amount of lignin and hemicellulose from this work tends to be lower than the work reported by^30,42^. This problem is very likely to occur due to the type of lignoselullosic biomass used^43^. We used OPEFB with very different lignocellulosic content, which suggests that the IL performance results could also be different. In addition, we suspect that the prepared OPEFB pulp (size =  ± 30 mesh) contributed to this result. The particle size, moisture content and type of biomass used can affect the pretreatment process^44^.

Furthermore, we pay serious attention to the use of the composition 91%. In this composition, the mass of IL [TEA][HSO_4_] is greater than that of 80% and 85%. The mass used in the composition of 91% is 20 g which correlates with the increasing number of [TEA]^+^ cation and [HSO_4_]^−^ anion. So that the OPEFB pretreatment conditions will present stronger acidic or alkaline properties. If we look again at Fig. 4C, we will find that the composition of 91% effectively reduces the lignin and hemicellulose content of OPEFB. This condition can be considered when pretreatment of OPEFB using IL [TEA][HSO_4_]. The reason behind this is the difference in cellulose content after pretreatment is not much different. Partial loss of cellulose can be due to the increased acidity of IL [TEA][HSO_4_]. In general, the acidic properties are reported to be related to the solubility of cellulose^45^. Another approach used to explain the solubility of cellulose during IL-based pretreatment is the type, geometry and size of anions, and the number of cations added. Cations are reported to interfere with oxygen atoms from glycosidic and hydroxyl through disperse forces and hydrogen bonding at the axial position of the cellulose fibers^46^.

**Figure 5:**
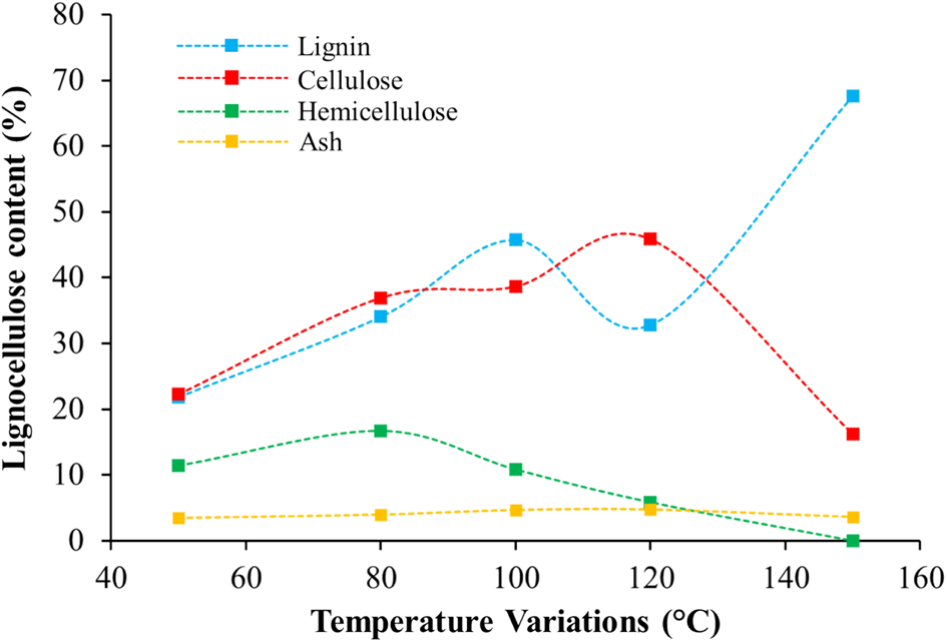
Optimization of OPEFB pretreatment time using IL [TEA][HSO_4_].

Figure 5 is the result of temperature optimization during OPEFB pretreatment using IL [TEA][HSO_4_]. Temperature optimization is another important step during the pretreatment process. The stubborn nature of lignin during the pretreatment process which can be transformed into other forms of lignin will greatly inhibit the performance of IL [TEA][HSO_4_]. It is necessary to release lignin so that the fermentation process is optimal. So this problem makes pretreatment conditions such as temperature as one of the variables that have an impact on lignin solubility. Several literatures report the use of variable pretreatment temperatures, as reported by^40^. This basis is what we use to study the effect of temperature (50, 80, 100, 120 and 150 °C) on IL performance in the OPEFB transformation.

On temperature optimization, our focus is on the total cellulose content. The general trend resulting from temperature optimization is that when the total amount of cellulose increases, there will be a decrease in the total amount of lignin and hemicellulose, and vice versa. This tendency was shown at the pretreatment temperature of 120 °C. We then report these results as the optimum temperature for pretreatment of OPEFB using IL [TEA][HSO_4_]. These results corroborate the work of the IL [TEA] [HSO_4_] application for biomass pretreatment, as reported by^42,47^. At this temperature, the total percentage for cellulose increase was 62.58%. As for the release of lignin and hemicellulose were 48.65% and 31.32%, respectively.

On the other side of Fig. 5, we observe how an increase in temperature causes a decrease in the total cellulose content and an increase in the total lignin content. This tendency occurs at a pretreatment temperature of 150 °C. In addition to the tendency for the deterioration of cellulose structure when the temperature increases, specifically for lignin it can be explained that there are non-lignin components that combine with lignin during pretreatment using IL [TEA][HSO_4_]. If we look at the data in Fig. 5, the non-lignin components are most likely cellulose and hemicellulose. Where at a temperature of 150 °C there was a significant decrease in both. This problem has also been previously reported by^40^. Another factor that causes the hydrolysis of cellulose and hemicellulose is none other than the acidic nature of IL [TEA][HSO_4_]. Both are hydrolyzed and then undergo a dehydration reaction to 5-HMF and furfural, and contribute to the condensation reaction.

### IL [TEA][HSO_4_] recovery

**Figure 6:**
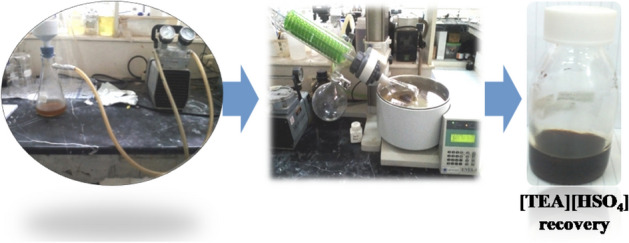
The recovery process of IL [TEA][HSO_4_].

Although several previous studies reported the ability of IL [TEA][HSO_4_] to be recovered and recycled, the different types of biomass make this process still interesting to study. Figure 6 shows how the IL [TEA][HSO_4_] recovery process we did. The recovery process goes through the same steps as reported by^30^ with the basic principle being a gradual washing by a solvent through the use of controlled temperatures. The temperatures we report in the recovery process are 80 °C and 100 °C. This temperature was not higher than the IL [TEA][HSO_4_] recovery temperature reported by^40^. Another application of IL [TEA][HSO_4_] has also been reported using temperatures greater than 100 °C^48^. Another consideration in selecting the recovery temperature is based on the results of our TGA analysis. Where the analysis results show that the decomposition temperature of IL[TEA][HSO_4_] is in the range 210 °C to 274.3 °C. So we assume that the choice of temperature of 100 °C does not change the properties of IL [TEA][HSO_4_]. The indicator of the success of our recovery process is the increasing density properties of IL [TEA][HSO_4_].

**Figure 7:**
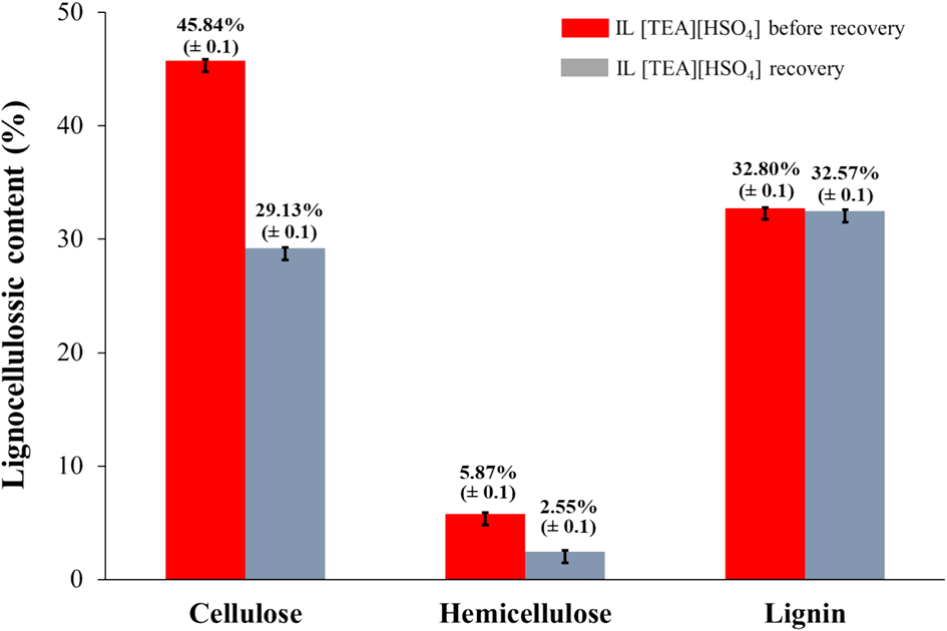
Comparison of pretreatment results between the IL synthesis and recovery.

In observing the performance of IL [TEA][HSO_4_] recovery in the OPEFB pretreatment process, we then compared it with our synthesized IL [TEA][HSO_4_]. The OPEFB pretreatment process uses IL [TEA][HSO_4_] recovery which we report based on the optimum conditions of synthesized IL [TEA][HSO_4_]. The difference in the results of the two is shown in Fig. 7. Referring to the results obtained, the performance of IL [TEA][HSO_4_] recovery was not optimal in increasing the total cellulose content and decreasing the total lignin content. We suspect that this is due to changes in the composition and properties of IL during OPEFB pretreatment. So that this is our priority in the future. However, when compared with data on lignocellulose content of OPEFB before pretreatment (Table 1) there was a change in the percentage of cellulose content from 27.40 to 29.13%. Likewise, the percentage of lignin content changed from 34.61 to 32.57%.

A comparison scenario of the OPEFB pretreatment process using IL and conventional methods is shown in Table 2. In addition to effectively releasing lignin and hemicellulose content, the application of ILs provides advantages, including low energy, short time and processing.

**Table 2:** Comparison of OPEFB pretreatment processes.

Pretreatment methods	Pretreatment stage	Optimization process
Combination of physics and chemistry	It is carried out in two steps: heating at high temperature and soaking using an alkaline solution	Heating temperature, alkaline concentration, OPEFB: alkaline ratio, and immersion time
Chemical solutions	Performed in two stages: deep immersion (i) alkaline solutions, and (ii) acid solutions	alkali and acid concentrations, immersion time for alkalis and acids, ratio of alkali/acid: OPEFB
ILs	Done in one stage (simultaneous stage)	ILs composition, temperature, pretreatment time, and reuse

## Conclusions

The ability of IL [TEA][HSO_4_] to transform OPEFB through pretreatment process was investigated in this work. This transformation aims to increase the cellulose content by reducing the lignin and hemicellulose content. The positive impact of our work is to facilitate the enzymatic hydrolysis of cellulose to reducing sugars such as glucose. In addition, this work is an attempt to increase the usefulness of IL [TEA][HSO_4_] in the pretreatment process. The optimization process shows that the selection of the IL composition and temperature are important factors in obtaining high cellulose content. The increase in cellulose content of OPEFB seen in the use of the IL [TEA][HSO_4_] composition was 85% with a pretreatment temperature of 120 °C. During the recovery process, the temperature must be kept constant. Changes in temperature can cause the decomposition of IL. These results form our basis in developing the pretreatment process of OPEFB in the future.
